# Angiopoietin-2 and Growth Differentiation Factor-15 as Predictors of Device-Detected Atrial Fibrillation Burden

**DOI:** 10.3390/biomedicines14040902

**Published:** 2026-04-16

**Authors:** Valentin Bilgeri, Philipp Spitaler, Jasmina Gavranovic-Novakovic, Theresa Dolejsi, Patrick Rockenschaub, Moritz Messner, Marc Michael Zaruba, Fabian Barbieri, Agne Adukauskaite, Markus Stühlinger, Bernhard Erich Pfeifer, Pietro Lacaita, Gudrun Feuchtner, Peter Willeit, Axel Bauer, Wolfgang Dichtl

**Affiliations:** 1 Department of Internal Medicine III, Medical University of Innsbruck, 6020 Innsbruck, Austria; valentin.bilgeri@i-med.ac.at (V.B.); jasmina.gavranovic-novakovic@tirol-kliniken.at (J.G.-N.); theresa.dolejsi@i-med.ac.at (T.D.); moritz.messner@tirol-kliniken.at (M.M.); marc-michael.zaruba@tirol-kliniken.at (M.M.Z.); agne.adukauskaite@tirol-kliniken.at (A.A.); markus.stuehlinger@tirol-kliniken.at (M.S.); axel.bauer@i-med.ac.at (A.B.); wolfgang.dichtl@i-med.ac.at (W.D.); 2 Institute of Clinical Epidemiology, Public Health, Health Economics, Medical Statistics and Informatics, Medical University of Innsbruck, 6020 Innsbruck, Austria; patrick.rockenschaub@i-med.ac.at (P.R.); peter.willeit@i-med.ac.at (P.W.); 3 Department of Cardiology, Angiology and Intensive Care Medicine, Charité—Universitätsmedizin Berlin, Corporate Member of Freie Universität Berlin and Humboldt-Universität zu Berlin, Hindenburgdamm 30, 12203 Berlin, Germany; fabian.barbieri@dhzc-charite.de; 4 Department of Cardiology, Angiology and Intensive Care Medicine, Deutsches Herzzentrum der Charité, Hindenburgdamm 30, 12203 Berlin, Germany; 5 Institute of Active Polymers and Berlin-Brandenburg Center for Regenerative Therapies, Helmholtz-Zentrum Hereon, 14513 Teltow, Germany; 6 Landesinstitut für Integrierte Versorgung Tirol, Tirol Kliniken, 6020 Innsbruck, Austria; bernhard.pfeifer@me.com; 7 Division for Digital Medicine and Telehealth, University for Health Sciences, Medical Informatics and Technology (UMIT-Tirol), 6060 Hall in Tirol, Austria; 8 Department of Radiology, Medical University of Innsbruck, 6020 Innsbruck, Austria; pietro.lacaita@tirol-kliniken.at (P.L.); gudrun.feuchtner@i-med.ac.at (G.F.); 9 Department of Public Health and Primary Care, University of Cambridge, Cambridge CB1 8RN, UK; 10 Ignaz Semmelweis Institute, Interuniversity Institute for Infection Research, 1090 Vienna, Austria

**Keywords:** device-detected atrial fibrillation, angiopoietin-2, growth differentiation factor-15, biomarkers, atrial fibrillation burden

## Abstract

**Background**: Pacemakers enable continuous long-term surveillance of atrial fibrillation detected by implanted devices. Circulating biomarkers reflecting endothelial dysfunction, inflammation, and myocardial stress may help identify patients at risk for atrial fibrillation (AF) progression and higher arrhythmic burden. **Methods**: This analysis included patients from the prospective ACaSA study (NCT05127720) with a dual chamber pacemaker (Microport^®^ BOREA DR or TEO DR) and monitored weekly via remote monitoring technology (SMARTVIEW^®^). Individuals with permanent AF or single-chamber systems were excluded. Baseline plasma concentrations of angiopoietin-2 (ANGPT2), growth differentiation factor-15 (GDF-15), fibroblast growth factor-23 (FGF-23), bone morphogenetic protein-10 (BMP10), and tumor necrosis factor–related apoptosis-inducing ligand receptor-2 (TRAIL-R2) were quantified using enzyme-linked immunosorbent assays. N-terminal pro-B-type natriuretic peptide (NT-proBNP) was measured using electrochemiluminescence immunoassay. Biomarkers were log_2_-transformed, with values below assay detection limits imputed at half the lower limit of detection. Two endpoints were assessed following a 30-day blanking period: (1) progression to persistent AF, defined as ≥7 consecutive days with >99% daily AF burden, analyzed using Cox regression; and (2) AF burden, calculated as total AF time normalized to monitored days and categorized as <25%, 25–75%, or >75%, analyzed using multinomial logistic regression. Multivariable models were adjusted for age, sex, heart failure, diabetes, and prior myocardial infarction; Cox models were limited to age, sex, and heart failure due to fewer events. **Results**: A total of 223 patients were included (median age 75 years; 37.2% women). During follow-up, 28 patients (13.3%) progressed to persistent AF. Higher baseline ANGPT2 was the strongest predictor of progression (HR per doubling 1.83, 95% CI 1.27–2.66, *p* = 0.001), followed by GDF-15 (HR 1.52, 95% CI 1.03–2.24, *p* = 0.036). In the burden analysis, ANGPT2 demonstrated a pronounced graded relationship with arrhythmic load, with markedly increased odds of high (>75%) AF burden (OR 8.31, 95% CI 2.63–26.26, *p* < 0.001). GDF-15 independently predicted both medium (OR 2.05, *p* = 0.025) and high burden (OR 2.32, *p* = 0.037). NT-proBNP displayed a borderline association with high burden (OR 2.02, *p* = 0.061). No significant associations were observed for FGF-23, BMP10, or TRAIL-R2. **Conclusions**: In continuously monitored pacemaker patients, ANGPT2 and GDF-15 emerged as key biomarkers associated with AF disease severity. ANGPT2 was strongly linked to both progression to persistent AF and high AF burden, whereas GDF-15 consistently predicted higher AF burden and also contributed to risk of progression. These findings highlight endothelial and inflammatory pathways as potential markers of atrial disease progression.

## 1. Introduction

Atrial fibrillation (AF) is the most common sustained cardiac arrhythmia and a major contributor to adverse cardiovascular outcomes. It is associated with substantially increased risks of stroke, heart failure, cardiovascular mortality, and all-cause mortality [1]. Beyond the mere presence of AF, higher AF burden—reflected by duration and frequency of atrial arrhythmic episodes—has been linked to progressively worse prognosis, underscoring the importance of not only detecting AF but quantifying its cumulative load [2,3].

Implantable pacemakers and other cardiac-implantable electronic devices permit continuous long-term rhythm monitoring in patients. Such devices provide a unique opportunity to detect subclinical or device-detected AF (DDAF) and to assess AF burden over several years—far surpassing intermittent surface ECG recording or ambulatory Holter monitoring. This capability allows for a more accurate and precise arrhythmia phenotyping, including timing, frequency and total burden of atrial arrhythmias [4].

Given this detailed monitoring capacity, there is growing interest in circulating biomarkers that might help predict the onset of AF or stratify patients according to their AF burden. Biomarkers reflect diverse pathophysiological pathways, including atrial stretch (volume/pressure overload), myocardial fibrosis, inflammation, endothelial dysfunction and atrial remodeling. For example, NT-proBNP is secreted by cardiomyocytes in response to wall stretch and left atrial volume overload and has been repeatedly associated with incident AF and AF recurrence [5,6,7,8]. NT-proBNP remains, however, essentially the only biomarker in widespread routine clinical use for AF risk prediction; other biomarkers have not yet been translated into everyday practice.

Mechanistically, biomarkers such as angiopoietin-2 (ANGPT2) and growth-differentiation factor-15 (GDF-15) implicate systemic, non-atrial pathways in AF. ANGPT2, an endothelial growth factor that antagonizes Tie2 signaling, promotes endothelial activation, vascular permeability, and inflammation, thereby contributing to vascular remodeling and pro-thrombotic endothelial dysfunction consistent with AF-associated stroke risk [9]. GDF-15, a member of the TGF-β superfamily, is upregulated in response to oxidative stress, inflammation, and mechanical strain, and has been linked to myocardial fibrosis and adverse cardiac remodeling [10,11,12,13].

Recent large biomarker studies extend these mechanistic insights to AF outcomes: in the AXAFA biomolecule study, higher baseline ANGPT2, BMP10 and NT-proBNP levels predicted early AF recurrence following catheter ablation, suggesting endothelial and shear-stress involvement [14]; likewise, in the EAST-AFNET 4 biomolecule study, elevated ANGPT2, BMP10, and NT-proBNP were associated with a lower probability of maintaining sinus rhythm during follow-up [15]. Together, these findings support that endothelial dysfunction and systemic stress signaling contribute to AF persistence beyond atrial-intrinsic mechanisms. However, no study yet has used DDAF incidence and burden as endpoints to assess the prognostic value of biomarkers.

In this context, the present study examines a “real-world” clinical cohort of pacemaker recipients (without permanent AF at baseline) equipped with continuous device-based monitoring, to evaluate the predictive value of multiple biomarkers for DDAF incidence and burden. This study aims to validate and extend previous biomarker research in the specific setting of continuous long-term monitoring via pacemaker devices, and to assess whether biomarkers reflecting different mechanistic pathways may help stratify risk of DDAF onset versus DDAF burden.

## 2. Materials and Methods

### 2.1. Study Cohort

Patients enrolled in the prospective observational ACaSA study (NCT05127720) and who were implanted with a Microport^®^ (Clamart, France) BOREA DR or TEO DR pacemaker formed the basis of the analysis. Participants were recruited at the Medical University Innsbruck after informed consent. All patients received telemedical monitoring of the pacemaker system using the SMARTVIEW^®^ (MicroPort CRM, Clamart, France) technology, transmitting data to the study center on a weekly basis. Only patients from the ACaSA Project with available baseline biomarker data were included. Patients with permanent atrial fibrillation (AF) at baseline were excluded from the analysis. To ensure reliable atrial sensing, patients with single-chamber (SR) devices were also excluded.

### 2.2. Biomarker Measurement

Biomarkers were collected at baseline during the patients’ initial visit for device implantation. Plasma concentrations of GDF-15, ANGPT2, FGF-23, BMP10 and TRAIL-R2 were quantified using commercially available enzyme-linked immunosorbent assay (ELISA) kits from different manufacturers: GDF-15 (Quantikine ELISA, R&D Systems, Minneapolis, MN, USA; Catalog No: DGD150), ANGPT2 (Quantikine ELISA, R&D Systems, Minneapolis, MN, USA; Catalog No: DANG20), FGF-23 (Invitrogen, Waltham, MA, USA; Catalog No: EH189RB), BMP10 (Cloud-Clone Corp., Houston, TX, USA; Catalog No: SEC106Hu), and TRAIL R2 (Merck, Darmstadt, Germany; Catalog No: RAB0481). NT-proBNP levels were measured using the Roche electrochemiluminescence immunoassay (Roche Diagnostics, Basel, Switzerland). Plasma samples previously stored at −80 °C were gently thawed at room temperature and prepared according to the respective manufacturer’s instructions for each assay. All measurements were performed in duplicate to ensure reproducibility. Appropriate standards, controls, and diluted plasma samples were added to 96-well plates pre-coated with analyte-specific antibodies and processed according to the manufacturer’s protocols. After incubation and washing, absorbance was measured using a Bio-Rad iMark microplate reader (Bio-Rad Laboratories, Hercules, CA, USA). Optical density was read at 450 nm for all assays, with wavelength correction at 570 nm for the GDF-15 and ANGPT2 assays, as recommended by the manufacturers.

### 2.3. DDAF

Device detected atrial fibrillation (DDAF) outcomes were measured using pacemaker-detected mode switch events. A mode switch happens when the atrial heart rate rises abnormally to over 120 beats per minute and is at least 25% faster than the previous eight beats. The device confirms this if either at least 28 of 32 beats or 36 of 64 beats are fast. Once triggered, the pacemaker records how long mode switching lasts, from 1 s up to 24 h per day.

A 30-day post-inclusion blanking period was applied to exclude post-procedural arrhythmias. We defined two primary endpoints to assess disease progression and total arrhythmic load:

- Progression to Persistent AF: A time-to-event endpoint defined as the first occurrence of ≥7 consecutive days with a daily AF burden > 99%. Patients who met this criterion during the 30-day blanking period were considered to have prevalent persistent AF and were excluded from the survival analysis.
- DDAF Burden: A categorical endpoint reflecting the cumulative time in AF over the entire monitoring period. For each patient, the total duration of AF was normalized by the number of monitored days. Burden was categorized based on the interquartile range: <25% (low), 25–75% (medium), and >75% (high).

### 2.4. Statistical Analysis

Baseline biomarker distributions were assessed for normality using the Shapiro–Wilk test and visual inspection of histograms. All biomarkers (NT-proBNP, FGF-23, ANGPT2, BMP10, TRAIL-R2, and GDF-15) exhibited right-skewed distributions and were log_2_-transformed. To account for left-censoring, values below the specific lower limit of detection (LOD) were imputed to half of the LOD prior to log-transformation. Effect estimates—hazard ratios (HRs) and odds ratios (ORs)—are interpreted per doubling of the biomarker concentration (i.e., per 1-unit increase on the log_2_ scale).

Associations with incident persistent AF (time to first episode of ≥seven consecutive days with >99% daily AF burden) were assessed using separate univariate and multivariable Cox proportional hazards models (Model 1). Patients meeting the persistent AF criteria during the blanking period were excluded from the survival analysis. Associations with DDAF burden were assessed using multinomial logistic regression (Model 2), categorizing burden as low (<25%), medium (25–75%), or high (>75%), with the low-burden group serving as the reference.

Multivariable models were adjusted for the following pre-specified covariates: age (per 5-year increment), sex, heart failure, diabetes mellitus, and history of myocardial infarction. Due to the lower number of events for the persistent AF endpoint, the multivariable Cox model (Model 1) was adjusted only for age, sex, and heart failure to avoid overfitting. Statistical significance was defined as a two-sided *p*-value < 0.05. All analyses were performed using R (R Foundation for Statistical Computing, Vienna, Austria).

## 3. Results

### 3.1. Study Population

A total of 223 patients were included in the final analysis. Median age was 75.0 years (IQR 68.0–80.0), and 37.2% were women. Primary pacing indications were sick sinus syndrome (50.2%) and atrioventricular block (43.5%). The prevalence of comorbidities was high, including arterial hypertension (65.0%), coronary artery disease (36.3%), and a history of clinical atrial fibrillation (40.4%). Baseline characteristics are shown in Table 1. The total number (N) of biomarkers varied slightly due to missing values (e.g., N = 207 for NT-proBNP), total N are presented in Table 2.

### 3.2. Biomarker Results

Biomarker measurements showed a median NT-proBNP concentration of 325.0 pg/mL [130.0–718.0], while median levels of ANGPT2, GDF-15, and FGF-23 were 2874.5 pg/mL [2102.0–3930.4], 1073.8 pg/mL [747.9–1656.4], and 0.6 pg/mL [0.3–41.5], respectively. TRAIL-R2 and BMP10 concentrations were comparatively lower, with medians of 2.5 ng/mL [2.5–3.2] and 0.2 pg/mL [0.2–0.7], respectively.

### 3.3. Model 1: Time to First Persistent AF

**Figure 1:**
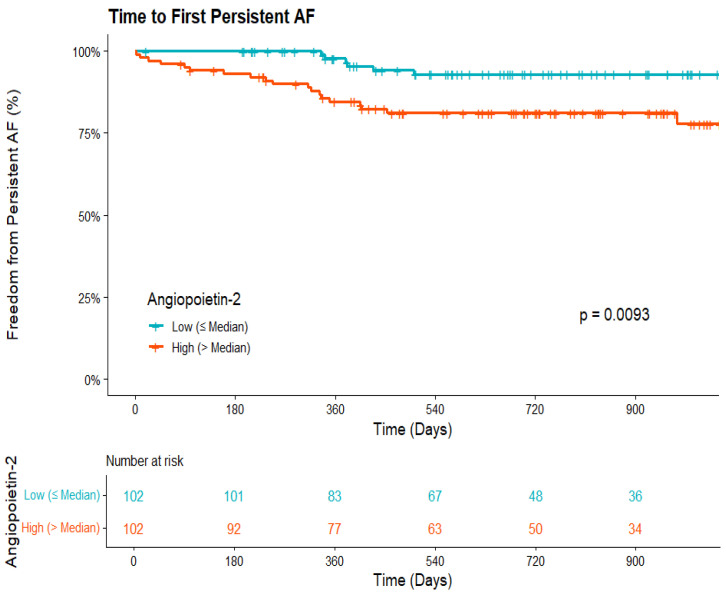
Kaplan–Meier curves depicting freedom from persistent DDAF stratified by median Angiopoietin-2 levels, with the number of patients at risk shown below the plot.

**Figure 2:**
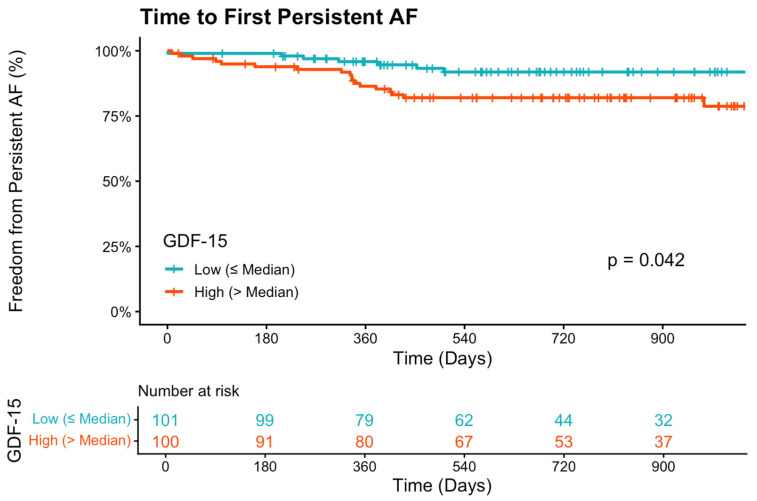
Kaplan–Meier curves depicting freedom from persistent DDAF stratified by median GDF-15-levels with the number of patients at risk shown below the plot.

**Figure 3:**
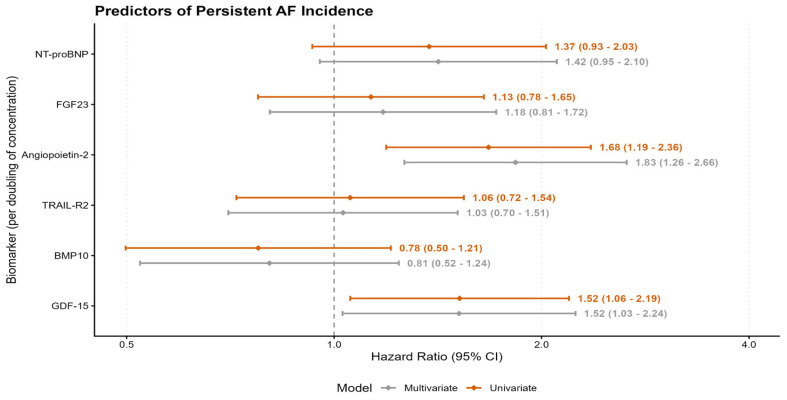
Forest plot displaying adjusted hazard ratios (HRs) with 95% confidence intervals for developing persistent AF per doubling of log_2_-transformed biomarker levels.

Of the 223 patients in the total cohort, 21 (9.4%) met the criteria for persistent AF during the 30-day blanking period and were excluded from the progression analysis. In the remaining at-risk cohort of 202 patients, 28 (13.9%) subsequently progressed to persistent AF during follow up. In multivariable Cox regression adjusted for age, sex, and heart failure, Angiopoietin-2 was the strongest independent predictor of progression. A doubling of baseline Angiopoietin-2 concentration was associated with an 83% increased risk of developing persistent AF (HR 1.83, 95% CI 1.27–2.66, *p* = 0.001, Figure 1). GDF-15 was also independently associated with progression (HR 1.52, 95% CI 1.03–2.24, *p* = 0.036, Figure 2). NT-proBNP showed a trend toward increased risk that did not reach statistical significance (HR 1.42, *p* = 0.085). Results of univariate and multivariable regression models can be seen in Table 2 and Figure 3.

### 3.4. Model 2: DDAF Burden

The multinomial regression analysis revealed strong associations for DDAF burden. (Table 3).

In the multinomial logistic regression analysis, Angiopoietin-2 exhibited a graded association with disease severity. While it was not significantly associated with medium burden (25–75%), a doubling of Angiopoietin-2 was associated with an 8-fold increase in the odds of having high (>75%) AF burden (OR 8.31, 95% CI 2.63–26.26, *p* < 0.001). GDF-15 was a consistent predictor across burden categories, significantly increasing the odds of both medium (OR 2.05, *p* = 0.025) and high burden (OR 2.32, *p* = 0.037). 

**Figure 4:**
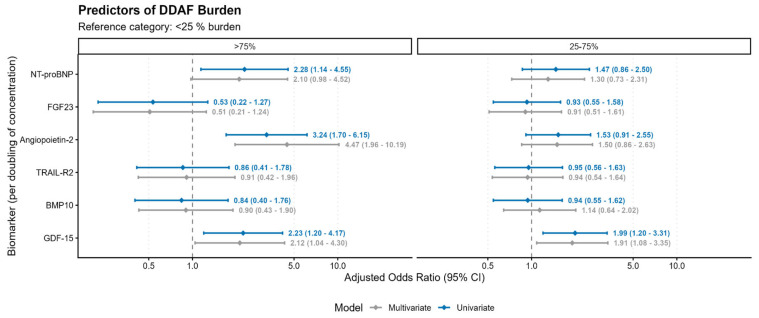
Forest plot showing adjusted odds ratios (ORs) with 95% confidence intervals for DDAF burden categories, comparing moderate (25–75%) and high (>75%) burden groups to the low burden group (<25%) across all biomarkers.

NT-proBNP showed a borderline significant association with high AF burden (OR 2.02, *p* = 0.061) (Table 3, Figure 4).

To evaluate the clinical utility of Angiopoietin-2 in identifying patients with the most severe arrhythmic phenotype, we performed ROC analysis for the high burden category (>75%). The biomarker demonstrated excellent discriminative power, with an Area Under the Curve (AUC) of 0.848 (95% CI 0.724–0.971). The optimal discrimination threshold was identified at 4303 pg/mL, yielding a high sensitivity of 89% and a specificity of 81%. This suggests that elevated Angiopoietin-2 levels above this threshold are highly indicative of extensive AF burden.

## 4. Discussion

In this prospective, continuously monitored pacemaker cohort, ANGPT2 was significantly associated with both the incidence and burden of device detected atrial fibrillation (DDAF). As a key novelty, we used continuous implantable device monitoring to define AF progression and quantify AF burden, enabling a precise assessment of their association with circulating biomarkers.

A doubling of ANGPT2 concentration was linked to an 83% higher risk of progression to persistent AF and more than an eightfold increase in the odds of belonging to the highest DDAF burden category. GDF-15 was also associated with progression to persistent AF as well as with DDAF burden, supporting the role of systemic stress and inflammatory signaling pathways in AF persistence. Notably, and in contrast to several large biomarker studies (without continuous monitoring), NT-proBNP and BMP10 were not significantly associated with DDAF incidence or burden in our cohort.

The role of biomarkers in AF pathophysiology and prognosis is an emerging field, which has been assessed in recent investigations. In an ARISTOTLE biomarker substudy, GDF-15 was robustly associated with stroke, bleeding, and mortality risk among patients with clinical AF [10]. More recently, the EAST-AFNET 4 biomolecule study demonstrated that lower levels of ANGPT2, BMP10, and NT-proBNP were associated with a higher probability of maintaining sinus rhythm during follow-up [15], while the AXAFA biomolecule study found that higher baseline levels of the same biomarkers predicted early AF recurrence after catheter ablation [14]. Together, these findings underscore the contribution of endothelial dysfunction, atrial wall stress, and shear stress to AF persistence, although the endpoints assessed in these studies differ substantially from those used in the present analysis.

Our findings reinforce the emerging role of endothelial activation markers, particularly ANGPT2, as powerful indicators of AF dynamics—even in a distinct population of patients requiring pacemaker implantation. The consistent association between ANGPT2, persistent AF and AF burden supports the concept that AF is not purely an atrial electrophysiologic disorder but is embedded in systemic endothelial dysfunction and subclinical inflammation. ANGPT2, a vascular growth factor within the angiopoietin/Tie (tyrosine kinase with immunoglobulin and EGF homology domains) signaling cascade, serves as a pivotal regulator of endothelial stability and vascular homeostasis. Under physiological conditions, it is stored in endothelial cells and contributes to the fine-tuning of vascular homeostasis as a counterpart to ANGPT1. During inflammation or endothelial injury, ANGPT2 is rapidly released into the circulation, acting as a context-dependent antagonist of Tie2 signaling [16]. This competitive inhibition disrupts endothelial barrier integrity, increases vascular permeability, and facilitates leukocyte extravasation and inflammation.

The resulting endothelial dysfunction provides a mechanistic link to atrial pathophysiology: by promoting acute electrophysiological alterations and, over time, inflammation triggered structural remodeling of the atrial myocardium.

The potential clinical relevance of ANGPT2 is further supported by our ROC analysis. ANGPT2 demonstrated excellent discriminative performance for identifying patients with high AF burden (>75%), with an AUC of 0.85. A threshold of approximately 4300 pg/mL yielded high sensitivity (89%) and specificity (81%). These findings suggest that ANGPT2 may function as a useful “rule-out” biomarker, identifying patients who are unlikely to develop extensive AF burden, potentially allowing for more personalized monitoring strategies.

Beyond individual biomarkers, our results align with a broader movement toward biomarker-informed risk stratification in atrial fibrillation, particularly in device-monitored populations. Recent work has demonstrated that validated risk scores combining clinical variables with circulating biomarkers can support personalized decision-making [17]. Within this framework, ANGPT2 and GDF-15 may provide complementary information to traditional clinical risk markers, helping to refine AF phenotyping and identify patients at risk for progressive or high-burden disease despite continuous monitoring.

The association of GDF-15 with AF burden aligns with its known link to oxidative stress and adverse cardiac remodeling. GDF-15 is a cytokine belonging to the transforming growth factor beta (TGF-ß) family. Although under physiological conditions GDF-15 is only weakly expressed in most tissues, its expression markedly increases in response to cardiovascular inflammation and tissue injury. This pattern suggests a potential contribution to AF progression through fibrotic, metabolic, and stress-related pathways at the myocardial level.

Contrary to previous large biomarker studies, our analysis did not reveal a significant effect of NT-proBNP or BMP10 on AF incidence or burden. Several mechanisms may explain this divergence. First, our cohort consisted exclusively of pacemaker patients –a distinct cohort with a specific profile (and a more advanced stage of disease?), many with underlying conduction disease or bradyarrhythmia—which may attenuate the relative influence of atrial stretch or volume overload on AF initiation compared to populations with structurally normal hearts or post-ablation patients. Second, continuous device-based monitoring allows for detection of short, subclinical AF episodes that may not generate sufficient hemodynamic or mechanical strain to induce NT-proBNP secretion. In contrast, prior studies typically focused on clinically manifest AF endpoints. Third, medication effects and the relatively stable hemodynamic state in a pacemaker population could further dampen the predictive value of natriuretic peptides and cardiac load biomarkers such as BMP10.

In addition, ventricular pacing burden may have influenced NT-proBNP levels independently of atrial fibrillation status. High percentages of ventricular pacing are known to induce ventricular dyssynchrony and increased myocardial wall stress, which can elevate NT-proBNP irrespective of atrial stretch. Given the heterogeneity of pacing indications in our cohort (e.g., sick sinus syndrome versus atrioventricular block), substantial variability in pacing percentages is likely and may have introduced additional noise, thereby obscuring a specific association between atrial stretch–related biomarkers and AF initiation.

Finally, the relatively small number of patients progressing to persistent AF limited statistical power to detect more modest biomarker effects; therefore, the absence of significant associations for BMP10 or TRAIL-R2 should be interpreted with caution rather than as evidence of no association.

### Strengths and Limitations

A key strength of this study lies in its continuous, high-resolution rhythm monitoring, which minimizes misclassification and allows precise quantification of DDAF burden—an advantage over conventional intermittent ECG-based approaches. The prospective real-world design provides valuable insight into the prognostic value of various serum biomarkers in a cardiac pacemaker population, reflecting everyday cardiology practice. In contrast, other studies have used other endpoints such as AF recurrence rate after catheter ablation or mortality. Our study is the first, investigating the association and prognostic value of biomarkers with AF burden in a very specific population—only in those requiring pacemaker implantation.

However, our study also has some limitations. The sample size was relatively small, limiting statistical power and potentially obscuring modest associations. In particular, the limited number of progression events (n = 28) reduced the ability to detect smaller effect sizes, favoring identification of strong predictors while potentially underpowering analyses of biomarkers such as BMP10 or TRAIL-R2. Furthermore, the restriction to a pacemaker population introduces selection bias towards older patients with more advanced disease including conduction disease, potentially limiting generalizability to broader AF, new-onset AF and other community cohorts. Finally, single baseline biomarker measurement precludes assessment of temporal biomarker dynamics relative to AF development.

## 5. Conclusions

In this continuously monitored pacemaker cohort, ANGPT2 and GDF-15 were strongly associated with device-detected AF burden, whereas NT-proBNP and BMP10 showed no significant associations. These findings highlight the importance of inflammatory endothelial and systemic stress pathways in AF progression and suggest that endothelial biomarkers may provide incremental prognostic information beyond traditional cardiac load markers in device-monitored patients. Larger studies are warranted to validate these findings and assess their clinical applicability.

## Figures and Tables

**Table 1:** Baseline characteristics for inclusion into the ACaSA study (n = 223). Results are reported as median [interquartile range] for continuous variables and as n (%) for categorical variables.

Characteristic	Median (IQR) or No. (%)
Age, years	75.0 [68.0–80.0]
Female sex, n (%)	83 (37.2%)
Body mass index, kg/m^2^	25.8 [22.7–28.4]
**Pacing Indication**	
Sick Sinus Syndrome	112 (50.2%)
AVB II (Wenckebach)	7 (3.1%)
AVB II (Mobitz)	32 (14.4%)
AVB III	58 (26.0%)
**Comorbidities**	
Arterial hypertension	145 (65.0%)
Diabetes mellitus	51 (22.9%)
Atrial fibrillation	90 (40.4%)
Heart failure	22 (9.9%)
Coronary artery disease	81 (36.3%)
Previous Myocardial Infarction	22 (9.9%)
**Medication**	
Beta blocker	63 (28.3%)
Statin	129 (57.8%)
ACE inhibitor or ARB	114 (51.1%)
Aspirin	75 (33.6%)
NOAC	74 (33.2%)
Loop-Diuretics	17 (7.6%)
**Cardiac Data**	
LVEF, %	58.0 [53.6–63.2]
Left atrial volume index (mL/m^2^)	32.5 [25.6–41.0]
LDL cholesterol (mg/dL)	85.0 [62.0–120.5]
**Biomarkers (Raw Values)**	
NT-proBNP (pg/mL)	325.0 [130.0–718.0]
Angiopoietin-2 (pg/mL)	2874.5 [2102.0–3930.4]
GDF-15 (pg/mL)	1073.8 [747.9–1656.4]
FGF-23 (pg/mL)	0.6 [0.3–41.5]
TRAIL-R2 (ng/mL)	2.5 [2.5–3.2]
BMP10 (pg/mL)	0.2 [0.2–0.7]

Abbreviations: ACE = Angiotensin-Converting Enzyme; ARB = Angiotensin Receptor Blocker; AVB = Atrioventricular Block; IQR = Interquartile Range; LDL = Low-Density Lipoprotein; LVEF = Left Ventricular Ejection Fraction; NOAC = Non-Vitamin K Antagonist Oral Anticoagulant; NT-proBNP = N-terminal pro-B-type Natriuretic Peptide.

**Table 2:** Hazard Ratio (HR) for developing Persistent AF per doubling of each biomarker concentration.

Biomarker	N	Univariate HR (95% CI)	*p*-Value	Multivariable HR (95% CI)	*p*-Value
Angiopoietin-2	217	1.68 (1.19–2.36)	0.003	1.83 (1.27–2.66)	0.001
GDF-15	217	1.52 (1.06–2.19)	0.025	1.52 (1.03–2.24)	0.036
NT-proBNP	207	1.37 (0.93–2.03)	0.111	1.42 (0.95–2.11)	0.085
FGF-23	217	1.13 (0.78–1.65)	0.522	1.18 (0.81–1.72)	0.396
TRAIL-R2	217	1.06 (0.72–1.54)	0.782	1.03 (0.70–1.51)	0.878
BMP10	215	0.78 (0.50–1.21)	0.262	0.81 (0.52–1.24)	0.328

**Table 3:** Odds Ratios (OR) for being in a higher DDAF burden category compared to the reference category with <25% AF burden.

Biomarker	Comparison (vs. <25%)	Univariate OR (95% CI)	*p*-Value	Multivariate OR (95% CI)	*p*-Value
Angiopoietin-2	25–75%	1.86 (0.91–3.79)	0.091	1.77 (0.81–3.85)	0.151
	>75%	5.26 (2.14–12.93)	<0.001	8.31 (2.63–26.26)	<0.001
GDF-15	25–75%	2.17 (1.24–3.81)	0.007	2.05 (1.10–3.83)	0.025
	>75%	2.46 (1.23–4.90)	0.011	2.32 (1.05–5.09)	0.037
NT-proBNP	25–75%	1.24 (0.94–1.64)	0.135	1.15 (0.85–1.56)	0.361
	>75%	1.57 (1.09–2.26)	0.016	1.49 (0.99–2.23)	0.055
BMP10	25–75%	0.98 (0.74–1.28)	0.851	1.07 (0.80–1.42)	0.658
	>75%	0.92 (0.64–1.33)	0.662	0.95 (0.66–1.38)	0.787
FGF-23	25–75%	0.98 (0.87–1.12)	0.799	0.98 (0.86–1.12)	0.743
	>75%	0.86 (0.71–1.06)	0.161	0.85 (0.69–1.05)	0.141
TRAIL-R2	25–75%	0.94 (0.53–1.67)	0.835	0.93 (0.52–1.69)	0.823
	>75%	0.84 (0.39–1.83)	0.663	0.90 (0.40–2.04)	0.802

## Data Availability

The data underlying this article cannot be shared publicly due to the privacy of individuals that participated in the study. The data will be shared upon reasonable request to the corresponding author.

## Abbreviations

The following abbreviations are used in this manuscript:

DDAF	Device detected atrial fibrillation
AF	Atrial fibrillation
ANGPT2	Angiopoietin-2
BMP10	Bone Morphogenetic Protein 10
ELISA	Enzyme-Linked Immunosorbent Assay
FGF-23	Fibroblast Growth Factor 23
GDF-15	Growth Differentiation Factor 15
NT-proBNP	N-terminal pro-B-type Natriuretic Peptide
TRAIL-R2	Tumor Necrosis Factor-Related Apoptosis-Inducing Ligand Receptor 2
